# Analysis of the Validity and Reliability of the Photo Finish^®^ Smartphone App to Measure Sprint Time

**DOI:** 10.3390/s24206719

**Published:** 2024-10-19

**Authors:** Luis Alberto Marco-Contreras, Ana Vanessa Bataller-Cervero, Héctor Gutiérrez, Jorge Sánchez-Sabaté, César Berzosa

**Affiliations:** Faculty of Health Sciences, Universidad San Jorge, Autov. A-23 km 299, 50830 Villanueva de Gállego, Spain; lamarco@usj.es (L.A.M.-C.); hgutierrez@usj.es (H.G.); osanchez@usj.es (J.S.-S.); cberzosa@usj.es (C.B.)

**Keywords:** mobile application, smartphone, validation, exercise fitness

## Abstract

In athletic training and research, the evaluation of sprint speed is widely used, and its accurate measurement is especially demanding. High-cost photocells are the gold-standard system for sprint time assessment, although low-cost smartphone applications can be a suitable option. This study assesses the validity and reliability of an application to measure sprint time compared to photocells. Five physically active subjects completed six sprints of 10 m and 20 m at maximal speed and a 5 m go and return sprint to evaluate the validity of the Photo Finish^®^ app (Version 2.30). To assess reliability, six trials of 5 m go and return sprints were measured by two smartphones. The validity results showed a mean bias of 0.012 s (95% CL: 0.000, 0.024) between the application and the photocells for the 10 m sprint, 0.007 s (95% CL: −0.007, 0.022) for the 20 m sprint and 0.005 s (95% CL: −0.005, 0.017) for the 5 m go and return test. The results also found R^2^ between both systems (R^2^= 0.9863, 0.990 and 0.958) for each distance (10 m, 20 m and 5 m go and return, respectively). As for reliability, the application showed outstanding consistency between two smartphones operating simultaneously (ICC 0.999; R^2^: 0.999). This study shows that the Photo Finish^®^ app is an accurate and reliable tool to measure sprint time with an error of 0.09 s.

## 1. Introduction

Sprinting ability is a determining factor in many sports disciplines, such as sprinting [1] and middle-distance running [2] in athletic events, as well as most team sports [3,4,5]. The ability to sprint differs according to the sport’s modality and its specific demands, mainly in terms of distance. However, previous studies seem to indicate that the variables related to sprinting ability are more individual than modality-dependent, which opens a range of possibilities for coaches to individualize training [6]. In addition, above a certain level, athletes may need years of training to obtain minimal improvements of the order of mere hundredths of a second, depending on the distance [7,8]. Therefore, the measurement and evaluation of sprint speed is widely used in the training and research field for competitive athletes in modalities where high-intensity running movements are performed [6]. Differences in performance in tests of such short duration are measured in very small units [7]. Therefore, most coaches and athletes use manual stopwatches for both training sessions and assessments. This system is accessible to anyone involved in sport, as it only requires a stopwatch. However, as it is highly observer-dependent, there is a greater probability of error, and differences of up to 0.19 s have been found between observers for the same distance [9]. In addition, differences can be found when comparing manual timing with the “gold-standard” methods, with an average difference of 0.31 s in the time recorded over distances of 40 yards (36.576 m) [9].

In recent years, some alternatives using high-speed video recording have been introduced. This method requires the athlete to be recorded during the race, including the start and finish, usually from a side view. The video is then analyzed using specific software. This system has shown high accuracy compared to photocell systems and an intraclass correlation coefficient (ICC) exceeding 0.98 [10]. The main disadvantage of this type of system is its inability to provide immediate results, something that can be essential for athletes during their training sessions. In order to solve this problem to some extent, very recently, mobile applications that, also based on high-speed video recording, make it possible to measure running time have been developed, with much faster analysis using the mobile device itself [11]. Again, this system has some disadvantages in terms of the need to place vertical visual references of start and end to allow for the exact determination of such moments and, therefore, for the time recording to be accurate. This process, in addition, later requires time for video analysis.

Recently, GPS devices have been used to evaluate speed and not only to assess distances. Different brands have developed “low-cost” units, showing a bias of 7,2% in velocity underestimation [12]. This bias could be sufficiently valid to measure load in football, but it is a problem in other disciplines, such as athletics, or when you are evaluating sprint time.

Consequently, each of the methods described above present different problems that impede their use as a regular tool for measuring sprint times. For this purpose, automatic measurement systems are considered the “gold standard” in terms of sprint time measurement and are used in official athletics competitions due to their reliability and accuracy [13]. For athletic events, World Athletics (formerly the International Association of Athletics Federations) has established a time measurement error of less than 0.001 s [14]. However, these systems are prohibitively expensive and impractical for routine use in training sessions or even for the assessment of most athletes.

There are other systems, such as photocell systems, which are also employed for this type of assessment and are considered highly reliable and accurate tools, provided certain aspects are standardized, such as the placement height of the different pairs of photocells [15]. However, both the high economic value and the space and time required for their setup mean that their use is practically limited to research or isolated sessions.

Based on these facts, there is a need for valid and reliable assessment tools and protocols that can detect changes in the performance of athletes with the necessary precision [16] while at the same time being simple to use and cost-effective so that the greatest number of people can have access to it.

The Photo Finish^®^ application (app) is a tool that uses the cameras of smartphone devices to detect the start and finish of an athlete performing a sprint. The system features an artificial intelligence program that detects when the athlete passes in front of the camera, starting or stopping the integrated stopwatch and instantly providing the recorded time. Furthermore, it is possible to connect several devices together, allowing for the measurement of not only the start and finish times but also partial times depending on the number of devices used. This is an important advance over the previously mentioned methods because it is a low-cost application (offering either a monthly subscription or a perpetual license) that can be used on the athlete’s or coach’s personal mobile device. It also showed good reliability in 100 m sprint [17], but there are no studies assessing its performance on shorter distances or in one-way-and-return tests. This could be really interesting for different sports where sprints are shorter, like team sports (football, basketball or handball). This study investigates both the accuracy and reliability of this application in time measurement and compares it with previously validated methods in different distances from 5 m to 20 m. The present research project seeks to determine the usefulness of the Photo Finish^®^ app as a tool for monitoring athletes both in their evaluation and in their training, by analysing the times obtained in different sprints.

## 2. Materials and Methods

### 2.1. Participants

Five subjects (four male, one female) recruited from the university staff participated voluntarily in this study. The participants’ age was 33 ± 6 years, with a mean height of 171 ± 5 cm and mean weight of 61.6 ± 7.5 kg. The inclusion criteria required participants to be physically active (at least 3 days of running activities weekly). Any subjects with a disabling injury in the previous month and/or with a cardiovascular disease were excluded from this study. The participants were informed about the measurement protocol and signed an informed consent document in compliance with the Declaration of Helsinki. The project was approved by the University Ethics Committee (93/1/21-22).

### 2.2. Design and Procedures

**Figure 1:**
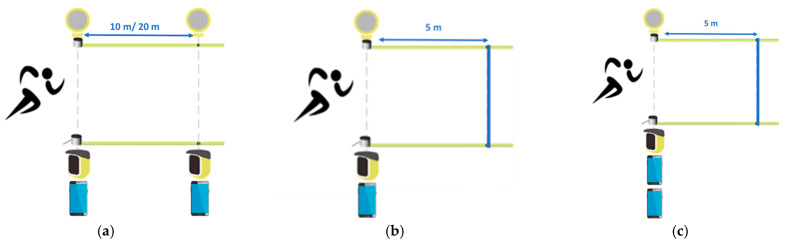
(**a**) Configuration for app validity assessment for a sprint in line test; (**b**) configuration for a go and return test; (**c**) configuration for app reliability assessment (Pictures adapted from https://training.microgate.it/en/products/witty/wittygate, accessed on 14 October 2024).

All the measurements were made in two days and one week apart. A running track was used in this study. Two pairs of dual beam photocell meters (Witty, Microgate, Bolzano, Italy) were placed on tripods at a height of 50 cm. These photocells offer an accuracy of 0.4 ms and a resolution on 0.125 ms according to the manufacturer. The photocell system technology is considered the reference in sprint time measurements [15]. Simultaneously, two smartphones with the Android operating system, Xiaomi One Note 10 (Xiaomi Inc., Beijing, China) and Xiaomi POCO X3 (Xiaomi Inc., Beijing, China), which had the Photo finish^®^ (version 2.30) app installed and were connected to each other were positioned over two tripods at the same location as the photocell emitters, although at a slightly lower height (Figure 1a). The Photo finish^®^ app works using the video camera of the smartphone (at 30 fps), detecting the moment the athlete crosses in front of the camera through artificial intelligence and activating or pausing a chronometer. The accuracy reported by the app designers is 20 ms.

The separation of the emitter and receiver photocell pair was 2 m, and the subject crossed at a meter from both the emitter photocell and smartphone. The distance between the photocell pairs was fixed at 10 m and at 20 m. For each distance, the participants completed 6 trials at maximal speed wearing clothing of a contrasting color compared to the background of the scene, as recommended by the manufacturer. The total number of sprint repetitions run by the participants was 30 for each distance analyzed. Moreover, a go-and-return measurement was tested using only one pair of photocells and one smartphone (Figure 1b). The distance between the line and the measurement system was 5 m. To assess the reliability of the Photo finish^®^ app, two smartphones were placed in the same location, and only one pair of photocells was used. The time to reach a line located at 5 m and return was considered in this assessment (Figure 1c).

### 2.3. Statistical Analysis

The results were described as mean (SD), and the Shapiro–Wilk test was applied to verify the normality of the data. A comparison of paired *t* tests was applied to compare the time measured with the photocell system and the app, with an adjusted R^2^ reported for each comparison. The validity of the smartphone application in comparison to the photocell system was analyzed by calculating the linear regression (R^2^) and performing a Bland–Altman analysis to determine systematic bias. The reliability of the Photo finish^®^ app was calculated by applying the linear regression (R^2^) and performing a Bland–Altman analysis between measurements of the app installed on two different mobile phones. Bias in Bland–Altman analysis was rated as trivial (<0.19), small (0.2–0.59), medium (0.6–1.19) or large (1.2–1.99). A two-way mixed-effects intraclass correlation coefficient for single measures (ICC) was included to describe how strongly devices resembled each other in validity and reliability measurements. An ICC value r > 0.9 was considered to indicate excellent agreement. The statistically significant level was established at *p* < 0.05 and the software used to perform the statistical calculation was GraphPad Prism (v9.4.1.681) and IBM SPSS Statistics (v28.0.1.0).

## 3. Results

### 3.1. Validity Outcomes

**Figure 2:**
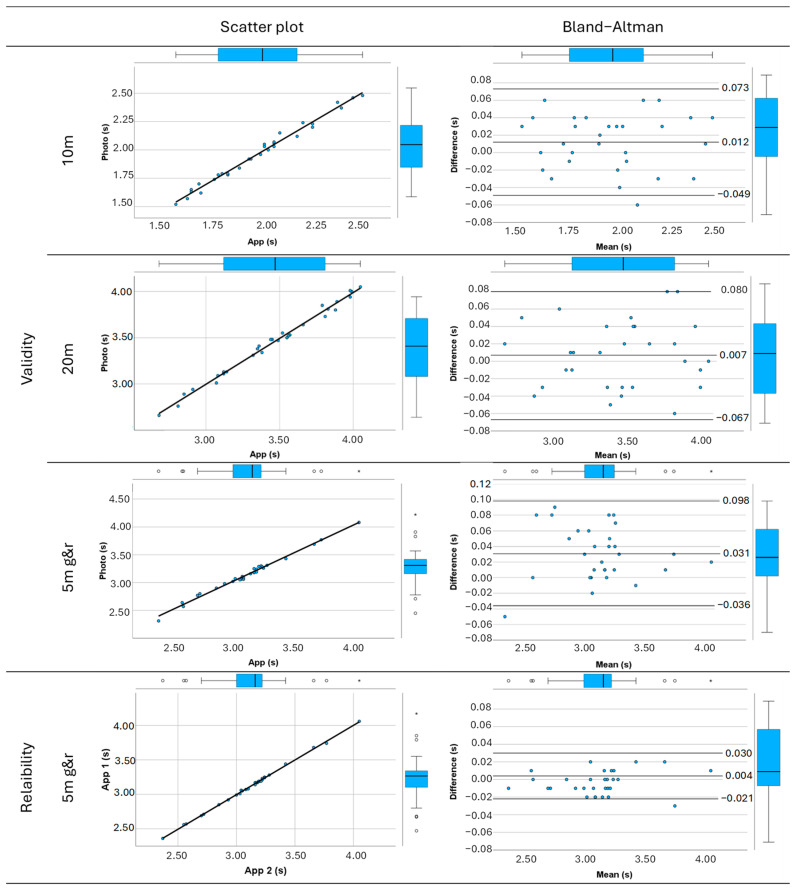
Validity: scatter plot with the linear regression of photocells (Photo) and the Photo finish^®^ app (App) and Bland–Altman difference plot vs. average of bias between photocells and the Photo finish^®^ app in 10 m separation, 20 m separation and 5 m go and return (g&r) trials. Reliability: scatter plot of the Photo finish^®^ app (App 1 and App 2) and Bland–Altman difference plot vs. average of bias between the Photo finish^®^ app (App 1 and App 2) in 5 m go and return (g&r) trials.

The results of the validity study of the Photo finish^®^ app are shown in Table 1. First, in the 10 m separation trial, a regression coefficient R^2^ = 0.986 and trivial bias between the app and photocell system measurements indicate that both systems express similar times. Comparing means with a *t* test shows a significant difference (*p* = 0.048) between the app and the gold-standard system, although the ICC is 0.992. As shown in the Bland–Altman plot, measurement errors (95% confidence limits: 0.000, 0.024) are distributed around the mean (0.012 s) (Figure 2).

Second, there is also a nearly perfect correlation and trivial bias in the 20 m separation trial, again indicating the validity of the Photo finish^®^ app in measuring time in a single sprint trial. No significant differences appear when comparing the means (*p* = 0.30). Figure 2 represents measuring errors (95% confidence limits: −0.007, 0.022) vs. mean (0.007 s) in a Bland–Altman plot.

Finally, when the measurement is only made with one mobile phone and one photocell in a 5 m go and return test, a nearly perfect correlation and trivial bias appear again. No significant differences are evident when comparing the means (*p* = 0.36). The Bland–Altman plot in Figure 2 shows a mean bias of 0.006 s (95% confidence limits: −0.005, 0.017).

Measurements in all trials have a small overestimation, showing a positive mean bias for each distance.

### 3.2. Reliability Outcomes

The results of the reliability study of the Photo finish^®^ app are shown in Table 1. Measurements made using two mobile phones in a 5 m go and return test show an ICC = 0.999 (95% confidence limits 0.998, 1.000) and R^2^ = 0.999. No significant differences are found when comparing the means (*p* = 0.14). The Bland–Altman plot shows a mean bias of 0.004 (95% confidence limits −0.001, 0.009), which is considered trivial bias, indicating great reliability (Figure 2).

## 4. Discussion

### 4.1. Principal Findings

The main objective of this study was to test the validity and reliability of the smartphone app Photo finish^®^ in measuring the 20 m sprint time and 5 m go and return sprint time compared to photocells as the reference measurement method. The main findings of this study are as follows: (i) The Photo finish^®^ smartphone app is a valid tool to assess time when compared to the gold standard (photocell timing gates), showing a mean bias of up to 0.012 s, ICC over 0.97 and R^2^ over 0.95 for each distance analyzed (10 m, 20 m and 5 m go and return). (ii) The Photo finish^®^ app has great reliability when the app’s results for the 5 m go and return test are compared between two smartphones, showing an ICC and R^2^ of 0.999. According to the Bland–Altman plots, the app shows high agreement with photocells for 10 m, 20 m and the 5 m go and return test, with trivial bias values (<0.19).

Surprisingly, a small overestimation was found when measuring times with the Photo finish^®^ app. There are other devices that have the same problem of indicating times that are a bit slower than the gold standard [18], and this is worth taking into account by trainers and athletes if they decide to use the app to measure sports performance.

A system for sprint time measurement should also be reliable. The Photo finish^®^ App shows an outstanding consistency between two smartphones operating simultaneously (ICC = 0.999).

Different devices have been used for measuring time in sprints [6]; however, the cost of these technologies is not always affordable for coaches or athletes. Low-cost and accessible solutions are being developed to solve this problem. Although radar or photocells are the reference systems for assessing time in sprints, the Photo finish^®^ app offers similar accuracy values for the 20 m test.

GPS is another technology validated for this purpose, also offering good accuracy in speed assessment [12,18]. These devices offer a correlation value of r = 0.97 with photocells for mean velocity in 40 m sprint, like that obtained in our study.

However, one of the main drawbacks of this technology, besides the cost, is the necessity of good visibility of satellites and, therefore, its functionality only applies in outdoor locations. Due to that, the use of smartphones for sprint time assessment emerges as an extremely viable solution.

The Photo finish^®^ app is not the only application on the market used for sprint time measurement. The MySprint app, developed by Pedro Jiménez-Reyes [14], has also been validated for 40 m sprint, obtaining a correlation coefficient of r = 0.989–0.999 between photocells and the app. Similar values have been reached for 20 m sprint (r = 0.995) in the current study. It has been observed that the correlation increases with the distance evaluated, as the time spent performing the sprint is higher, so the relative error is lower. The MySprint app has been validated for 40 m sprint, with partial times measured at 5, 10, 15 and 20 m, indicating a value of r between 0.989 and 0.999.

The main difference between both studies is the type of device used for validation: the MySprint was validated for iPhones, while the present study used two Android devices. Our study also analyzes the reliability of the Photo finish^®^ app by comparing measurements from two different devices in the 5 m go and return, showing a very high correlation between measurements (bias mean −0.004, 95% CL: −0.001, 0.009, *p* = 0.14). In contrast, the reliability study of the MySprint app also shows high reliability (mean difference 0.004, SD 0.03, *p* = 0.999), but it was calculated based on the differences found between observers, not between devices.

Another study validated an app for Change of Direction (COD) time [19]. In the study, the application was compared with photocells and stopwatches in a COD of 5 m. This test is like the 5 m go and return conducted in our study. The results found a concurrent validity with timing gates, with an ICC of 0.999 (95% CL: 0.998, 1.000) and R^2^ = 0.999.

These findings make the Photo finish app (at the lowest frame rate, i.e., 30 fps) a useful tool for controlling the evolution of running times. It has an error of 0.09 s (maximal mean difference between the gold standard and the app), which is higher than the error at the frame rate stated by the manufacturer (0.02 s). The video recording used to identify when athletes cross the “finish line” operates at a velocity of 30 fps, which limits the accuracy of the app. This means that the app may potentially miss an event occurring within this interval, thus reducing the accuracy of the measurement. For this reason, the app is not recommended when a higher accuracy is needed, such as in World Athletics events, where the error must be less than 0.001 s [14], at least with this low frame rate of 30 fps.

### 4.2. Limitations

Despite these promising results, this study presents limitations.

One limitation may be the use of Android devices only, which excludes iOS devices. Although the app may function in a similar way, it could be interesting to evaluate whether the mobile phone brand or model could affect its validity and reliability.

A second limitation of the current study is the restricted scope of reliability testing across devices. The reliability of the app was only tested for the 5 m go and return test due to technical constraints. Thus, these findings might be limited. We do not yet have evidence to suggest that the high level of inter-device reliability found in this test would be consistently achieved across other types of tests or distances.

### 4.3. Future Research

In future studies, the use of four devices (two at the start and two at the finish line) would help to further explore and verify the application’s reliability in measuring a sprint without return. Moreover, additional research could be conducted to further optimize and fine-tune the application’s accuracy beyond the current 10 ms constraint due to the video recording speed.

Given the increasing trend of integrating artificial intelligence and machine learning technologies into mobile applications, these advancements could be utilized to enhance the app’s capabilities, accuracy and user experience. Furthermore, this kind of application could also be created and validated for other sports and fitness parameters beyond sprinting, expanding its potential uses.

## 5. Conclusions

This study shows very good agreement between the smartphone application analyzed and the photocell taken as a reference method for 10 m and 20 m sprint and a 5 m go and return test.

The Photo finish^®^ application offers an accurate and reliable low-cost tool for coaches and athletes to assess sprint performance using only a smartphone in a reliable way, considering the error in measurement.

## Figures and Tables

**Table 1:** Validity and reliability of the Photo finish^®^ app in 10 m, 20 m and 5 m go and return (g&r) trials. All parentheses include the lower and upper limits of the 95% confidence interval. Mean bias: mean of the absolute error between paired measurements; CV bias: mean bias SD/gold standard mean; ICC: two-way mixed-effects intraclass correlation coefficient for single measures; R^2^: adjusted R^2^; SEE: standard error of the estimate.

								Regression Analysis			*t* Test	
		n		Mean (s)	Mean Bias (s)	CV Bias	ICC	R^2^	*p*	SEE	*p*	Hedge’s g
Validity	10 m	30	Photo	1.971 (1.869, 2.072)	0.012 (0.000, 0.024)	1.617 (1.208, 2.027)	0.992 (0.983, 0.996)	0.986	<0.001	0.032	0.048	0.043 (−0.001, 0.088)
			App	1.982 (1.882, 2.084)								
	20 m	30	Photo	3.447 (3.303, 3.591)	0.007 (−0.007, 0.022)	1.106 (0.826, 1.386)	0.995 (0.990, 0.998)	0.990	<0.001	0.039	0.301	0.018 (−0.018, 0.055)
			App	3.543 (3.310, 3.599)								
	5 m g&r	29	Photo	4.773 (4.718, 4.828)	0.006 (−0.005, 0.017)	0.620 (0.460, 0.779)	0.978 (0.954, 0.990)	0.958	<0.001	0.028	0.295	0.039 (−0.037, 0.114)
			App	4.779 (4.726, 4.831)								
Reliability	5 m g&r	30	App_1	3.112 (2.982, 3.241)	0.004 (−0.001, 0.009)	0.427 (0.319, 0.536)	0.999 (0.998, 1.000)	0.999	<0.001	0.013	0.141	0.010 (−0.004, 0.024)
			App_2	3.115 (2.987, 3.244)								

## Data Availability

The original contributions presented in the study are included in the article, further inquiries can be directed to the corresponding author.
